# Topical Biocomposites Based on Collagen, Hyaluronic Acid and Metronidazole as Periodontitis Treatment

**DOI:** 10.3390/ph17101336

**Published:** 2024-10-07

**Authors:** Madalina Georgiana Albu Kaya, Alice Geanina Simonca, Ileana Rau, Alina Elena Coman, Minodora Maria Marin, Lacramioara Popa, Roxana Trusca, Cristina-Elena Dinu-Pirvu, Mihaela Violeta Ghica

**Affiliations:** 1 Department of Collagen, National Research and Development Institute for Textiles and Leather—Division of Leather and Footwear Research Institute, 93 Ion Minulescu Str., 031215 Bucharest, Romania; albu_mada@yahoo.com; 2 Faculty of Chemical Engineering and Biotechnology, National University of Science and Technology Politehnica Bucharest, 1-7 Gh. Polizu Street, 011061 Bucharest, Romania; simoncaalice@gmail.com (A.G.S.); ileana.rau@upb.ro (I.R.); roxana_doina.trusca@upb.ro (R.T.); 3 Department of Physical and Colloidal Chemistry, Faculty of Pharmacy, “Carol Davila” University of Medicine and Pharmacy, 6 Traian Vuia Str., 020956 Bucharest, Romania; lacramioara.popa@umfcd.ro (L.P.); cristina.dinu@umfcd.ro (C.-E.D.-P.); mihaela.ghica@umfcd.ro (M.V.G.); 4 Innovative Therapeutic Structures Research and Development Centre (InnoTher), “Carol Davila” University of Medicine and Pharmacy, 6 Traian Vuia Str., 020956 Bucharest, Romania

**Keywords:** metronidazole, collagen sponges, hyaluronic acid, sustained drug release, periodontitis, freeze-drying

## Abstract

Background: It is well known that periodontitis affects the gums and surrounding connective tissue. The chronic inflammatory response induced by bacteria in the gingival tissue leads to the loss of the collagen connection between the tooth and the bone and ultimately to bone loss. Methods: In this context, the aim of this research was the obtaining and characterization of a drug release supports in the form of sponges based on collagen, hyaluronic acid as a support and metronidazole as an antibiotic for the treatment of periodontitis. The sponges were characterized by FT-IR spectroscopy, water uptake, contact angle, SEM microscopy, in vitro metronidazole release analysis from sponges and data modeling. Results: The results showed that all the sponges had a porous structure with interconnected pores, the pore sizes being influenced by hyaluronic acid and metronidazole; the spongious structure became much more dense for samples with metronidazole content. All metronidazole-loaded sponges showed good surface wettability and an adequate swelling capacity for a suitable antimicrobial release at the periodontal pocket. The porous structures allow a controlled release, fast in the first hour, essential to control the initial microbial load at the periodontal level, which continues slowly in the following hours to ensure an effective treatment of periodontitis. Conclusions: Correlating all physical–chemical and bio-pharmaceutical results obtained, a promising solution for periodontitis treatment could be a met-ronidazole–collagen–hyaluronic system consisting of 1% collagen, 1.5% metronidazole and 0.8% hyaluronic acid, and in vitro and in vivo tests are recommended to continue studies.

## 1. Introduction

Periodontitis affects the gingiva and surrounding connective tissue and represents a cellular inflammatory disease that destroys these tissues and bones that support teeth. The connective tissue, the periodontium, consists of four components: the cementum, the periodontal ligament (PDL), the alveolar bone, and the gingiva. Chronic adult periodontitis appears due to bacterial accumulations on teeth. The bacterially induced chronic inflammatory response in the gingival tissue leads to a loss in collagen connection between the tooth and the bone, and finally to bone losses [1,2,3].

The severe form of chronic periodontitis is a globally widespread pathology of the human oral cavity in the adult population, and affects almost 11% of the worldwide population, making it the sixth most prevalent disease of humanity [4,5,6]. Studies have shown that between 2011 and 2020, periodontitis impacted roughly 60% of the population, with about 24% of those suffering from the severe form. These studies demonstrate an extremely significant growth when compared to forecasts from 1990 to 2010 [7,8,9].

According to severity and progression, the present diagnostic classification of periodontitis is split into four phases and three grades. An appropriate periodontal therapy modality can be used once a patient’s case has been properly classified.

Nowadays, non-surgical and surgical treatments, paired with pharmacological adjuncts are used in conventional periodontal therapies [10,11,12]. Scaling, root planning, and antibiotics are examples of treatments that may be used if the periodontitis is not advanced. Dental surgery, including flap surgery, soft tissue grafts, bone grafts, guided tissue regeneration, and tissue-stimulating proteins, could be necessary if the periodontitis is advanced [13,14,15].

Antibiotics treat infections by eradicating or stunting the growth of bacteria and other organisms, but they have many side effects, from minor allergic reactions to serious, incapacitating adverse outcomes, when ingested. Local treatments with antibiotics can be used in addition to oral therapy to prevent secondary effects of the latter [16,17,18,19].

Current antibacterial local therapy, commercially available, can be applied by the patient at home, or in a dental clinic. These local forms of antibacterial drug therapy can also be divided into non-sustained, sustained and controlled release systems. The active agent in the non-sustained systems is released immediately; supra and sub-gingival irrigation solutions are included here. On the other hand, sustained systems contain a high concentration of active agent released for an extended period. In order to provide a safe, efficient, long-lasting, and convenient drug delivery impact, sustained drug release systems, represent a prolonged and slow drug release. Biocomposites based on polymers are highly investigated for sustained release of an encapsulated active ingredient, especially hydrophilic and hydrophobic polymers [20,21], and core–shell structures [22].

The last category, controlled release systems, includes semi-solid systems (chips, films, strips), fibers, and nano or microparticle systems that incorporate the active ingredient in the delivery medium and are placed in the periodontal pocket by an expert in a dental office; these systems will gradually release adequate concentrations of the active ingredient over a period longer than 24 h [23,24,25,26].

According to scientific results obtained so far, this paper presents a new biomaterial, a sustained drug release system, with collagen, hyaluronic acid and metronidazole for periodontitis local treatment. The collagen we propose for this study is type I and of bovine origin, the most abundant type in the organism [27]. It is well known that the periodontium contains collagen and hyaluronic acid, leading to a better acceptance of this proposed composite by the organism.

Collagen represents the primary component and support for the periodontium. Collagen constitutes 60% of gingival tissues, 70–80% of the periodontal ligament, and 90% of the alveolar bone matrix [28,29,30]. Another important component that exists in all periodontal tissues is hyaluronic acid in different amounts. Gingiva and the periodontal ligament are more abundant in hyaluronic acid than cement and alveolar bone are [31]. The antibacterial and anti-inflammatory properties of hyaluronic acid lead to its use in periodontitis treatments [32].

The scientific literature presents many systems for locally applied periodontitis treatment, but none of them include collagen, hyaluronic acid and metronidazole in the same medical device. For example, for guided tissue regeneration (GTR), blended hydrogels composed of polyvinyl alcohol and fish collagen were prepared as GTR membranes [33] and for nano-system treatment, metronidazole-loaded electrospun polylactide stereo complex nanofiber mats were successfully fabricated using the electrospinning process for treatment of periodontal disease [34]. Also, nanofibers with trilayer eccentric side-by-side structures, obtained through multiple-fluid electrospinning, represent a potential candidate to treat periodontitis [35].

According to our previous studies, the designed collagen hydrogels with metronidazole in different concentrations preserved the collagen triple helix structure, while the addition of strontium ranelate in the formulation led to a structural alteration. The kinetic parameters, the cumulative drug released percentage and the diffusion coefficient for the collagen hydrogels containing only metronidazole are influenced by the drug concentration, the best results being obtained for the sample containing 1.5% metronidazole-medium concentration level [36]. Also, other studies proved that an MTZ concentration higher than 2% induces an increase in the hydrogel viscosity, leading to a resistance of the drug diffusion through the polymeric network.

The aim of this research was to obtain and characterize some spongious collagen–hyaluronic acid supports with metronidazole in order to evaluate the physical–chemical, structural, and morphological properties as well as the drug deliverability in order to be used as a biomaterial for periodontitis.

## 2. Results and Discussion

The samples of collagen sponge with and without HA and MTZ were evaluated by morphology, structure and properties of support for drug delivery and the results are presented below.

### 2.1. FT-IR Spectroscopy for M1–M6 Sponges

**Figure 1:**
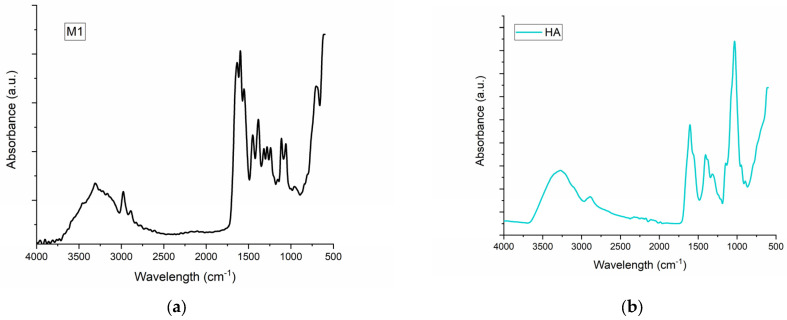

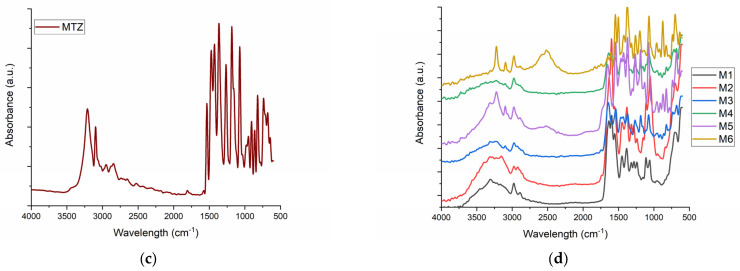
The FT-IR spectra for (**a**) M1 collagen; (**b**) HA-HA; (**c**) MTZ-MTZ; (**d**) the spongious matrices M1-M6.

The FT-IR for M1–M6 samples and for the main components of samples are presented in Figure 1.

The FT-IT spectra for collagen control (Figure 1a) show Amide A and B at 3309 and 2889 cm^−1^, respectively, and Amide I, II and III at 1635, 1554 and 1451 cm^−1^ as our previous results showed for collagen. HA presents a broad band around 3272 cm^−1^ which is assigned to the O-H groups and a peak at 1606 cm^−1^ associated with C-O stretching of carboxylate anion [37]. The control sample of MTZ (MTZ) showed a characteristic vibrational peak for C-H stretching at 3211 cm^−1^. The peaks at 1586 and 1530 cm^−1^ were assigned to C=C and C=N stretching, respectively. The N=O asymmetric stretching was assigned to peak at 1473 cm^−1^, and the C-C stretching peak was assigned to peak at 1428 cm^−1^. The absorption peaks at 1364 cm^−1^ were assigned to CH_3_ bending and N=O asymmetric stretching, respectively. Further, absorption peaks at 1265, 1073 and 738 cm^−1^ were assigned to C-O stretching, C-N stretching, and =C-H bending, respectively. FT-IR data of control MTZ was well supported by the literature data [38]. The influence of MTZ on the samples is more evident in the samples which contain HA. The FT-IR proved that MTZ does not change the triple helical collagen structure as our previous results showed [36] but that there is a clear interaction between MTZ and HA, as the water absorption and contact angle showed. The characteristic vibrational peak for C-H stretching at 3223 cm^−1^ is very highlighted for samples M4 and M6, meanwhile for samples M3 and M5, which contain the same amount of MTZ, it is visible at the same intensity. Also of interest is the peak of 2400 cm^−1^, which can be assigned to new crosslinking, more intense for the M6 than for the M4 sample and does not appear for the other sample. Comparing FT-IR spectra of M6 and M4, that peak is due to the higher presence of MTZ in sample M6.

### 2.2. Scanning Electron Microscopy (SEM) of M1–M6 Sponges

**Figure 2:**
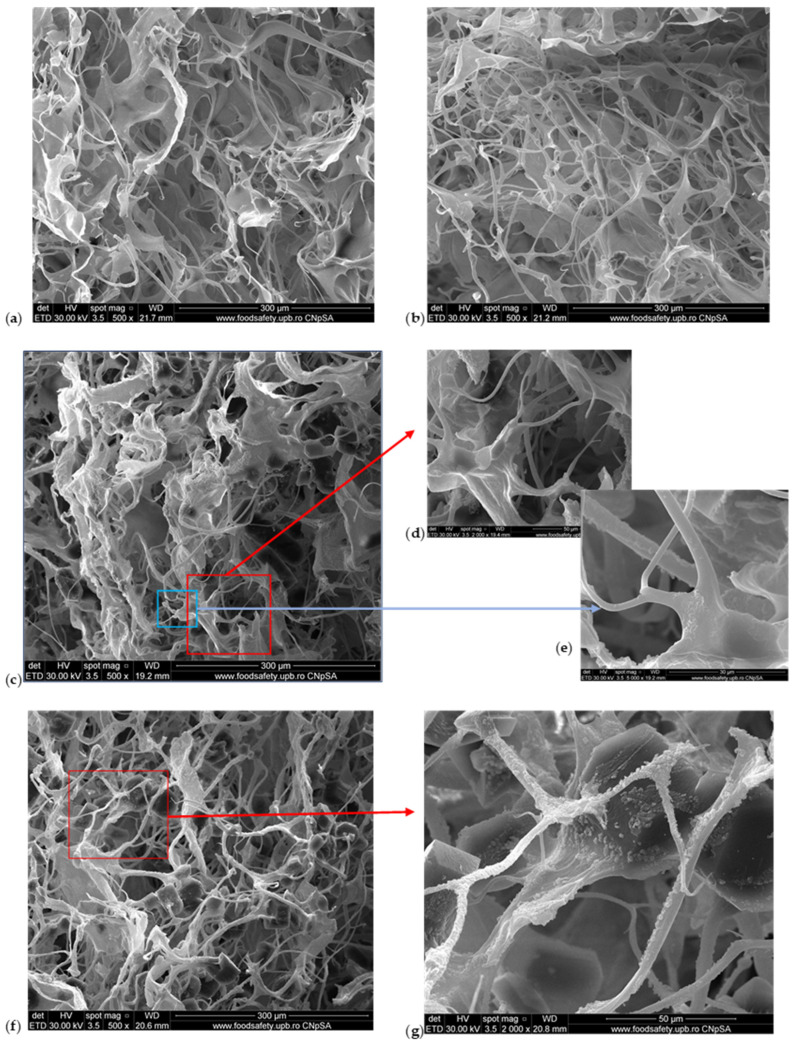

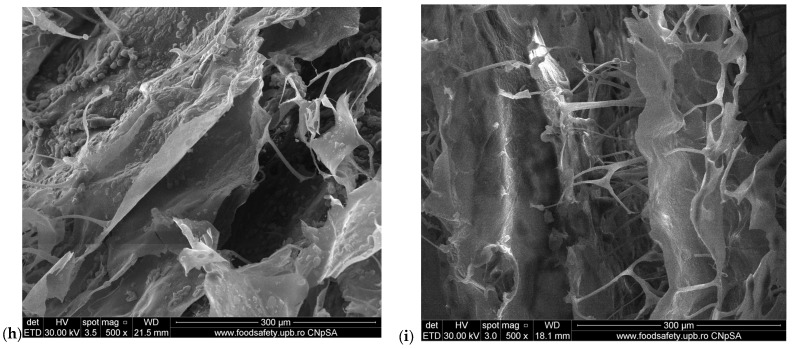
SEM images of M1–M6 sponges: (**a**) M1 (×500 magnification); (**b**) M2 (×500 magnification); (**c**) M3 (×500 magnification); (**d**) M3 (×2000 magnification); (**e**) M3 (×5000 magnification); (**f**) M4 (×500 magnification); (**g**) M4 (×2000 magnification); (**h**) M5 (×500 magnification); (**i**) M6 (×500 magnification).

The SEM microscopy presents the spongious structure with interconnected pores for all the samples as Figure 2 shows. The control sponge, M1, showed a structure based on fibrils which form pores with sizes between 50 and 150 µm (Figure 2a). The addition of HA forms a structure with higher porosity and smaller pore sizes, the majority being about 100 µm (Figure 2b). Figure 2c proves the compact structure when the MTZ is added. At higher magnitudes (×2000 in Figure 2d and ×5000 in Figure 2e), the presence of MTZ on collagen fibrils is clearly visible. The M4 sponge looks like the most homogenous sample (Figure 2f); this can be due to the presence of hyaluronic acid. The pore sizes become smaller than the others without MTZ or HA and the MTZ is still visible on the fibrils of collagen.

The water absorption showed that the M5 and M6 samples became more compact, and less absorbent because of the higher amount of MTZ. This is visible by SEM in Figure 2h,i where the MTZ is in excess, the porous structure is very compact, and the size and form of pores are not so clear anymore.

### 2.3. Water Uptake Ability of M1–M6 Sponges

**Figure 3:**
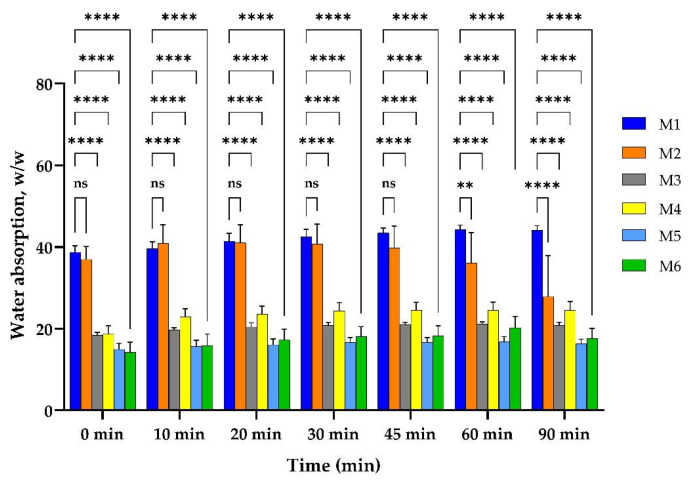
Water uptake ability of M1–M6 matrices. Results are not statistically significant (ns) for *p*-value > 0.05; results are statistically significant for *p*-value as follows: ** *p*  <  0.01, **** *p*  <  0.0001.

The water absorption results are presented in Figure 3 for all samples during 90 min, being at equilibrium after that period of time.

The controlled sample, M1, which contained only crosslinked collagen, uptook over 38 g/g water from the first moments and reached equilibrium at about 44 g/g, after one hour. The sample M2, which contained both collagen and HA, uptook about 37 g/g water in the first minutes, reached equilibrium in 10 min, and kept it for half an hour; then, the samples lost their weight in a slow degradation. A high statistical significance could be seen for samples that contained MTZ, all of which reduced their capacity of water absorption to less than half, uptaking about 18 g/g in the first minutes. The samples with a higher amount of MTZ uptook a smaller amount of water. This is because the MTZ is a lipophilic drug [39]. On the contrary, the samples with HA uptook a little bit more water than the ones without HA. Compared to the M1 matrix, it can be seen that the capacity to absorb the water of the M2 sample has a small variation in the first 45 min, followed by a significant decrease in the water uptake ability at 60 and 90 min. The samples with MTZ exhibit a highly significant decrease in the water absorption ability compared to the M1 sample, this behavior being related on the one hand to the presence of the drug and on the other hand to the presence in the same sample (M4 and M6) of the HA.

### 2.4. Contact Angle Evaluation of M1–M6 Sponges

The wetting behavior of the porous structure surface is important in different fields [40] including the medical one [41], and particularly for the design of spongious materials used as drug delivery supports [42]. The contact angle method was applied to determine the surface wetting ability of the sponges, with a lower contact angle value indicating a high hydrophilicity of the sponge surface [43]. The hydrophilicity is an important property facilitating the interaction between the sponge and biological environment [42], allowing both a rapid surface wetting and imbibition of the gingival crevicular fluid (GCF) within the drug release support. Due to the small values reported for the GCF, up to 8 μL under healthy conditions and up to 44 μL under inflammatory periodontal conditions [44,45], adequate sponge surface wetting is crucial for the penetration and diffusion of the dissolution liquid into the porous matrix structure, favoring the drug release from the supports.

**Figure 4:**
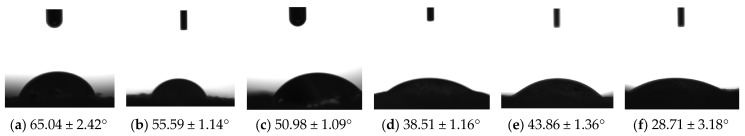
Images of the drop shape and the corresponding contact angle mean values for the spongious matrices: (**a**) M1; (**b**) M2; (**c**) M3; (**d**) M4; (**e**) M5; (**f**) M6.

In Figure 4 are illustrated examples of the drop shape images for the contact angle recorded for the tested sponges.

As can be seen from Figure 4, the sponge composition is reflected in the contact angle values obtained. The values recorded for the contact angles for all formulations were lower than 90^o^, ranging from 28.71 ± 3.18 (M6) to 65.04 ± 2.42° (M1). The highest value for the contact angle was noticed for the collagen sample without MTZ and HA, and the smallest one for the sample with a maximum concentration of drug and HA. The addition of HA (M2) in the formulation leads to a CA decrease of about 1.17 times reported in M1.

Analyzing the results obtained for the sponges M1, M3, and M5 based on collagen and MTZ, it can be seen that the presence of the drug at the inferior level in sample M3 leads to a CA decrease of about 1.27 times compared to M1, while the MTZ concentration of 2% generated a decrease of about 1.48 times. In the series M2, M4, and M6 with the presence of HA in the sponge formulation, a much higher decrease was observed, namely approximately 1.45 times for the M4 sponge (1.5% MTZ), and 1.94 times for the M6 sponge (2% MTZ), respectively, compared with the M2 sponge. One can remark that the presence of both MTZ and HA determined a higher hydrophilicity of the sponge surface, more obvious for a higher MTZ concentration. Comparing the samples M3 and M4 with 1.5% MTZ, and M5 and M6 with 2% MTZ, respectively, it can be observed that the CA value is more influenced by the presence of HA in the formulation (a decrease of about 1.34–1.2%), while evaluating sponges M3 and M5 and M4 and M6 ones, a higher drug concentration leads to a smaller CA decrease of approximately 1.16–1.32%. MTZ, a lipophylic drug, may not fully dominate the surface characteristics due to its dispersion within the collagen or HA matrix. Additionally, HA, which is known for its hydrophilic properties, likely offsets the hydrophobic tendencies of MTZ by enhancing the porosity and facilitating better surface wetting. This interplay results in the sponges showing improved wettability despite the presence of a hydrophobic drug.

On the other hand, it is known that the nature of porosity, such as pore size, distribution and interconnectivity, affects the contact angle. Smaller interconnected pores allow the liquid to imbibe more quickly, reducing the contact angle further. In contrast, larger or more isolated pores may slow down liquid penetration, resulting in higher initial contact angles [40,46]. Corroborating the results from the contact angle determination and the analysis of pore morphology using SEM analysis, it can be concluded that the inclusion of hyaluronic acid was found to increase the sponge’s porosity, which corresponded to a significant decrease in contact angle values. This suggests that HA determined a more porous structure, and facilitated quicker liquid absorption, leading to a lower contact angle.

The obtained results showed a suitable hydrophilicity of all sponge surfaces. The combination of MTZ with other more hydrophilic materials, like HA, can overcome its hydrophobic nature, making the resulting material more suitable for biomedical applications. In the particular case of periodontal application, an adequate sponge surface wettability, as a critical key point for fluid absorption, facilitates the penetration of the gingival crevicular fluid into the porous structure and the subsequent diffusion of the drug through the polymer matrix architecture.

### 2.5. In Vitro Drug Release Analysis from Sponges and Data Modeling

An important aspect of the design and evaluation of antimicrobial-loaded topical biocomposite intended for periodontitis treatment is the investigation of the drug release characteristics.

**Figure 5:**
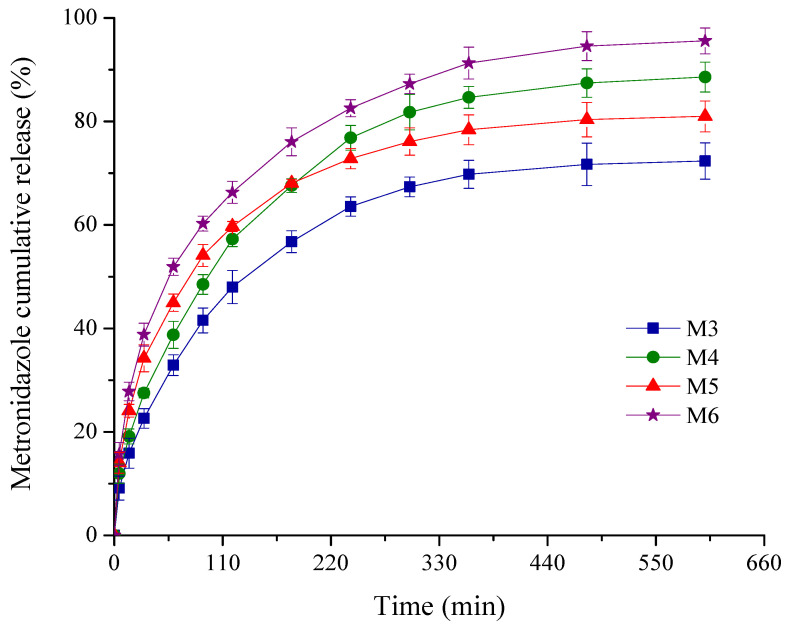
Plot of % cumulative drug release profiles from the sponges versus time.

Figure 5 illustrates the cumulative release profiles of the MTZ from the sponges in phosphate buffer pH 7.4 selected as in vitro release medium, compatible with the gingival fluid pH in periodontitis [47].

As depicted in Figure 5, comparable kinetic patterns are obtained for the M3-M6 sponges with a variable composition in MTZ and HA. Thus, the drug delivery process was described by two different phases: an initial rapid release in the first 60 min, followed by the second stage of a slower and progressive release for the next 9 h.

For antimicrobial topical delivery, the biphasic kinetic profiles are targeted both for prophylactic and treatment of a periodontal infection [48,49,50]. The burst release effect is important to control the initial microbial load at the periodontal level, providing a rapid effect and attaining minimum inhibitory concentration (MIC) [50,51]. Among the pathogen bacteria incriminated in periodontitis, in accordance with the literature data, the least susceptibility was reported for the *Actinobacillus actinomycetemcomitans* (*Aggregatibacter actinomycetemcomitans*) [51]. This is the reason why, in the present work, we have chosen two higher MTZ concentrations to obtain a rapid burst release more than MIC of periodontal pathogenic bacteria. It is noticed that for the designed sponges with MTZ M3-M6, the released drug concentration after the first hour of the experiment was superior to the MIC reported [52] for the above periodontopathogen bacterium. The lowest burst release of 32.89% was obtained for the sponge M3 with lower MTZ concentration and without HA, while the highest value (51.91%) was recorded for the sponge M6 with higher MTZ concentration and HA.

The gradual and sustained MTZ release in the next hours further ensures the prevention of bacteria multiplication or invasion. With respect to the cumulative drug released percentage after 10 h of experiment, the sponges could be graded in the following order: M6 (95.57%) > M4 (88.59%) > M5 (80.98%) > M3 (72.36%). Comparing the results obtained for the samples M3 and M4, and M5 and M6, respectively, it seems that the drug released (%) is affected by the presence of HA to a greater degree, an increase of about 1.20 times being recorded for the sponges M4 and M6, while the increase in the MTZ concentration in the sponge formulation has a smaller influence (Table 1).

Related to different reported studies, Kida et al. [52] performed an in vitro MTZ release from porous matrices, based on gelatin and two cellulose derivatives (carboxy methylcellulose and hydroxyethyl cellulose), over a period of 2 h. A burst release effect was noticed for all samples, more obvious in the first 20 min for the hydroxyethyl cellulose-based matrices. After an MTZ-loaded matrix single intra-pocket application, positive clinical results were obtained, indicating the local administration of the designed formulations as a promising alternative to the systemic one.

Panday et al. [53] described some MTZ microemulsion-loaded hydrogel for periodontitis prevention. The kinetic experiments were conducted over a period of 5 h, the released drug percentage varying from about 40 to 80%. The in vitro and ex vivo outcomes showed that the designed formulations could be effective in periodontitis treatment.

Hoang Nhan Ho et al. [54,55] reported that the MTZ release from different formulations (MTZ SLN-loaded HEC gel, MTZ SLNs and MTZ-loaded HEC gel) presented a considerable burst release effect, the amount of the drug delivered at 2 h varying from 61.41 to 91.05%. Moreover, the free drug (MTZ powder) was released more than 90% within 1 h. The MTZ incorporated free form in HEC gel was about 100% released after 3 h, while approximately 100% of the MTZ amount was released from the MTZ SLNs and MTZ SLN-loaded HEC gel at 24 h.

Besides the aforementioned works, different studies with MTZ-based formulations with a drug release extended more than 24 h were reported. Barat et al. [50] described chitosan inserts presenting an initial burst release of about 80% recorded at the end of 24 h (approximately 20% drug released in the first 4 h), independent of the formulation factors, followed by a sustained MTZ release in the next 6 days. In their paper, Jitrangsri et al. [55] developed an MTZ-loaded in situ forming matrix, using camphor as a matrix-forming agent. A variable burst release effect was noticed ranging from 20 to 80 in the first 6 h, followed by a sustained MTZ delivery reaching a maximum between 1.5 and 6 days, depending on the cosolvents. In another study, Khan et al. [56] developed biodegradable chitosan films based on MTZ and levofloxacin for periodontitis treatment. The cumulative MTZ release (%) was about 80% for the non-crosslinked films within 24 h, while for the crosslinked ones, it was less than 60%. After 24 h, the release was sustained and progressive up to 2–7 days depending on the crosslinking degree.

In this regard, it could be considered that our designed MTZ-loaded collagen sponges balanced well the effect of an initial rapid drug release with a gradual delivery for a longer period of time, essential for local antimicrobial therapy in periodontitis.

To predict the drug transport mechanism, different kinetic models were used, including the Higuchi, zero-order and power law models. The two primary metrics used to evaluate the goodness of fit of the kinetic models are the correlation coefficient (R) and adjusted R squared (R^2^). Adjusted R^2^ represents a relevant indicator when comparing the previous three models because, in addition to the evaluation of the goodness of fit, it also penalizes the kinetic models for their complexity. This aspect is essential when these three mathematical models are compared regarding their different complexities: the power law model involves more parameters (the release exponent n) than Higuchi and zero-order [57]. The R and adjusted R^2^ values for all MTZ-loaded sponges according to the above model are listed in Table 1. The values obtained for the correlation coefficients showed that the drug release was best described by the power law model, with the R highest values ranging between 0.9841 and 0.9897, and the adjusted R^2^ highest values varying from 0.9622 to 0.9757. The power law model presented the highest R and adjusted R^2^ values compared to those of Higuchi and zero-order models, which means that this mathematical model is regarded to fit the experimental data better, considering the complexity of the model.

Moreover, we compared these kinetic models using a tool called Akaike Information Criterion (AIC) that is precisely designed to compare the models rather than a goodness of fit approach, which is best described by R and adjusted R^2^. The AIC method generates numerical values which indicate both accuracy (goodness of fit) and precision (model variability) [58]. Considering the small number of experimental points, for kinetic analysis, the small-sample corrected AIC (AIC_c_) was used. Thus, the model that best statistically represents the mechanism of the drug release is the one with the AIC_c_ lowest value [59]. As can be seen in Table 1, the smallest values of AIC_c_ are recorded for the power law model, which is regarded as a better fit for the data, taking into account the complexity of this model. Correlating all of these, the highest R and adjusted R^2^ and the lowest AICc values were used to select the kinetic model with the best fit of the experimental data, which was the power law model.

The kinetic parameters specific to the power law model, namely release exponent and kinetic constant, are listed in Table 1. The release exponent values are smaller than 0.5 (varying from 0.31 to 0.37), indicating a deviation from the Higuchi model due to the complex processes that occur when the sponge comes into contact with the periodontal pocket. Initially, the drug retained onto or near the sponge surface (according to SEM images) is rapidly desorbed at contact with the crevicular gingival fluid, and then the absorption of the release medium in the porous structure takes place, followed by the polymer hydration and sponge swelling. This stage is associated with the burst release effect and is favored by an adequate sponge wetting ability, supported by the values of the contact angle smaller than 90° (varying between 28.71 and 50.98°), as well as by a suitable absorption in the first hour (Figure 1). Further, the diffusion-controlled release of the drug entrapped in the collagen fibrils (also concordant to images obtained from SEM analysis) takes place through the pores or channels within the polymer network, simultaneously with the gradual disintegration of the support [49,52]. This phase corresponds to the MTZ gradual and sustained release over the next 9 h of the experiment. We proposed for future investigations to use simulated crevicular gingival fluid in order to study the hydrophily, biodegradability and ability to release the drug.

## 3. Materials and Methods

### 3.1. Materials

Type I fibrillar collagen was extracted from calf hide using the technology currently available at the INCDTP-Division Leather and Footwear Research Institute—Collagen Department [60] with an initial concentration of 2.85% and acidic pH. Metronidazole (MTZ) was supplied from Hubei Hongyuan Pharmaceutical Technology Co., Ltd., Wuhan, China. Glutaraldehyde (GA) and hyaluronic acid (HA) were purchased from Sigma-Aldrich (Germany). All the experiments were carried out using ultra-pure water.

### 3.2. Methods

#### 3.2.1. Preparation of Sponges by Freeze-Drying Hydrogels

The initial collagen gel (2.85%) was adjusted to a 1% (*w*/*v*) concentration and a pH of about 7.4 with 1 M sodium hydroxide solution and distilled water under mechanical stirring. Different proportions of MTZ and HA were added to the gel and then all the samples were crosslinked with 0.2% glutaraldehyde. The composition of hydrogels is presented below in Table 2.

To obtain new sponges, the hydrogels presented in Table 2 were crosslinked, poured into glass Petri dishes with 5.2 cm diameter and left for 24 h at 4 °C and then the hydrogels were freeze-dried for 48 h using a Delta 2–24 LSC (Martin Christ, Osterode am Harz, Germany) as we previously described [61]. All the samples were used for further characterization, which is described below.

#### 3.2.2. Fourier-Transform Infrared Spectrometry (FTIR) of Sponges

FTIR spectra were performed using a Vertex 70 Bruker FTIR spectrometer (Billerica, MA, USA) with an attenuated total reflectance (ATR) module. For the obtained samples, the FTIR spectra were recorded in the ATR-FTIR mode at a resolution of 4 cm^−1^ in 600–4000 cm^−1^ wave number region.

#### 3.2.3. Scanning Electron Microscopy of Sponges

SEM analyses were performed on an FEI, Inspect F50 electron microscope equipped with a Schottky Field Emission source and an EDS detector, in the primary electron beam, on samples covered with a thin gold layer.

#### 3.2.4. Water Uptake of Sponges

For the water absorption determination, the collagen samples were placed in water and maintained at a temperature of 37 °C. At fixed time intervals, the samples were withdrawn and weighed. The water absorption was determined using the equation (Equation (1)):

(1) $$\%Water\;uptake=\frac{Wt-Wd}{Wd}\times 100(g/g)$$

where *Wd* represents the weight of the dry samples and *Wt* is the weight of the swollen samples at immersion time t. The experiments were conducted in triplicate.

#### 3.2.5. Sponge Contact Angle Evaluation

The sponge surface properties were monitored through contact angle (CA) measurements, performed with a KSV Cam 101 Scientific Instrument, and the protocol was reported in our previous work [42,62]. Measurements were taken on smooth, representative areas to minimize the effects of porosity and surface irregularities. Young equation’s (Equation (2)) was used for the contact angle evaluation:

(2) $$\gamma_{S}{=\gamma }_{\mathrm{SL}}{+\gamma }_{L}\cos\theta$$

where γ_S_ represents the surface tension of the solid, γ_SL_ is the solid/liquid interfacial tension, γ_L_ is the surface tension of the liquid, and θ is the contact angle made by the liquid drop with the solid porous surface. Contact angles were recorded immediately after droplet deposition to reduce liquid penetration. The initial contact angle was used for analysis. A high-speed camera (5 frames/16 ms) captured the droplet behavior, using the FAST method for precise evaluation. The experiments were carried out in triplicate.

#### 3.2.6. In Vitro Drug Release Analysis from Sponges and Data Modeling

The drug release characteristics from the designed sponges were investigated using a sandwich device adapted to dissolution equipment in conjunction with paddle stirrers (Esadissolver), in accordance with the method previously detailed [48]. Aliquots withdrawn at defined time points (5, 15, 30, 60, 90, 120, 180, 240, 300, 360, 480, and 600 min) were collected from the release vessel, and the MTZ content in each sample was spectrophotometrically evaluated (Perkin Elmer UV-Vis Spectrophotometer) at its maximum absorbance associated with a wavelength of 319 nm [36]. Each release experiment was performed in triplicate.

The % cumulative release profiles as a function of time were built. To analyze the release mechanism, the experimental data were fitted to the general power law model (Equation (3)):

(3) $$\frac{m_{t}}{m_{\infty }}=k\times t^{n}$$

where m_t_/m_∞_ represents the fraction of MTZ released at time t, k is the release kinetic constant (min^−1^) considering the geometric and structural traits specific to the dosage form, and n is the release exponent (dimensionless) showing the drug delivery mechanism. Two particular cases of this kinetic model, zero-order (n = 1) and Higuchi time square root model (n = 0.5), respectively, were also applied. For the Higuchi model, the drug diffusion rate through the polymeric network is much lower than that of polymer relaxation, while for the zero-order, the release rate is independent of time, and the polymer relaxation rate becomes the determining factor of the overall drug transport. When n has different values than 0.5 and 1, both rates are comparable, and the drug release is related to other processes which happen simultaneously [63,64].

#### 3.2.7. Statistical Analysis

Statistical analysis was carried out with GraphPad Prism 10.3.1, TableCurve 2D vs. 01, and Excel 2016 software. The experimental data were declared as means ± standard deviations (SDs). Multiple comparisons between the groups were realized employing two-way ANOVA (analysis of variance) with a 0.05 significance level Dunnett test. The obtained results were considered statistically significant for *p*-values less than 0.05.

## 4. Conclusions

Periodontitis has a lot of complications such as tooth and bone loss and many researchers are focused on developing new materials to prevent and treat it. In this context, collagen sponges with different metronidazole and hyaluronic acid concentrations intended for periodontitis treatment were designed and characterized. The sponges presented good surface wettability and an adequate swelling capacity for a suitable antimicrobial release at the periodontal pocket. The modulation of the MTZ and HA concentrations in the sponge formulation could balance the aforementioned characteristics well. FT-IR spectroscopy data show that the influence of metronidazole is more evident in samples containing hyaluronic acid. The recorded FTIR spectra demonstrated that the presence of metronidazole does not change the triple helical structure of collagen, as shown by previous results, but there is a clear interaction between metronidazole and hyaluronic acid, as shown by the results regarding water absorption capacity and hydrophilic–hydrophobic balance. SEM microscopy shows the spongious structure with interconnected pores for all samples. The presence of metronidazole is clearly visible on the collagen fibrils and forms a denser structure. The drug was fast released in the first hour, essential to control the initial microbial load at the periodontal level, followed by a gradual and sustained release of metronidazole over the next hours ensuring further prevention of bacterial multiplication or invasion. The designed spongious forms could be a viable alternative for periodontitis treatment and a starting point for new metronidazole-based formulations. Correlating all physical–chemical and biopharmaceutical results obtained, a promising solution for periodontitis treatment could be a metronidazole–collagen–hyaluronic system consisting of 1% collagen, 1.5% metronidazole and 0.8% hyaluronic acid, and in vitro and in vivo tests are recommended to continue studies.

## Figures and Tables

**Table 1:** Correlation coefficients for different kinetic models; release kinetics parameters characteristics for the power law model and cumulative MTZ released percentage.

Sponges	Zero-Order Model			Higuchi Model			Power Law Model			Release Exponent	Kinetic Constant (1/min)	MTZ Released (%)
	R	Adj R^2^	AIC_c_	R	Adj R^2^	AIC_c_	R	Adj R^2^	AIC_c_			
M3	0.8701	0.7085	−46.18	0.9698	0.9290	−64.53	0.9841	0.9622	−72.74	0.37	0.077	72.36
M4	0.8796	0.7286	−42.07	0.9742	0.9390	−61.48	0.9862	0.9672	−69.55	0.37	0.089	88.59
M5	0.8295	0.6260	−41.27	0.9504	0.8842	−56.51	0.9845	0.9633	−71.47	0.30	0.131	80.98
M6	0.8511	0.6695	−38.97	0.9621	0.9109	−56.02	0.9897	0.9757	−72.90	0.31	0.141	95.57

**Table 2:** Composition of hydrogels (quantities of Coll, HA and MTZ are reported for 100 g gel).

Sample Code	Coll, %	MTZ, %	HA, %	GA, %
M1	1	0	0	0.2
M2	1	0	0.8	0.2
M3	1	1.5	0	0.2
M4	1	1.5	0.8	0.2
M5	1	2.0	0	0.2
M6	1	2.0	0.8	0.2

## Data Availability

Data is contained within the article.
